# Glycoinositolphospholipids from *Leishmania braziliensis* and *L. infantum*: Modulation of Innate Immune System and Variations in Carbohydrate Structure

**DOI:** 10.1371/journal.pntd.0001543

**Published:** 2012-02-28

**Authors:** Rafael Ramiro Assis, Izabela Coimbra Ibraim, Fátima Soares Noronha, Salvatore Joseph Turco, Rodrigo Pedro Soares

**Affiliations:** 1 Centro de Pesquisas René Rachou, Fundação Oswaldo Cruz - FIOCRUZ, Belo Horizonte, Brazil; 2 Departmento de Microbiologia, Universidade Federal de Minas Gerais, Belo Horizonte, Brazil; 3 Department of Biochemistry, University of Kentucky Medical Center, Lexington, Kentucky, United States of America; Hebrew University-Hadassah Medical School, Israel

## Abstract

The essential role of the lipophosphoglycan (LPG) of *Leishmania* in innate immune response has been extensively reported. However, information about the role of the LPG-related glycoinositolphospholipids (GIPLs) is limited, especially with respect to the New World species of *Leishmania*. GIPLs are low molecular weight molecules covering the parasite surface and are similar to LPG in sharing a common lipid backbone and a glycan motif containing up to 7 sugars. Critical aspects of their structure and functions are still obscure in the interaction with the vertebrate host. In this study, we evaluated the role of those molecules in two medically important South American species *Leishmania infantum* and *L. braziliensis*, causative agents of visceral (VL) and cutaneous Leishmaniasis (CL), respectively. GIPLs derived from both species did not induce NO or TNF-α production by non-primed murine macrophages. Additionally, primed macrophages from mice (BALB/c, C57BL/6, TLR2−/− and TLR4−/−) exposed to GIPLs from both species, with exception to TNF-α, did not produce any of the cytokines analyzed (IL1-β, IL-2, IL-4, IL-5, IL-10, IL-12p40, IFN-γ) or p38 activation. GIPLs induced the production of TNF-α and NO by C57BL/6 mice, primarily via TLR4. Pre incubation of macrophages with GIPLs reduced significantly the amount of NO and IL-12 in the presence of IFN-γ or lipopolysaccharide (LPS), which was more pronounced with *L. braziliensis* GIPLs. This inhibition was reversed after PI-specific phospholipase C treatment. A structural analysis of the GIPLs showed that *L. infantum* has manose rich GIPLs, suggestive of type I and Hybrid GIPLs while *L. braziliensis* has galactose rich GIPLs, suggestive of Type II GIPLs. In conclusion, there are major differences in the structure and composition of GIPLs from *L. braziliensis* and *L. infantum*. Also, GIPLs are important inhibitory molecules during the interaction with macrophages.

## Author Summary

*Leishmania infantum* (*syn. L. chagasi*) and *L. braziliensis* are the causative agents of VL and CL, respectively, in the New World. A vital part of the parasite's life cycle involves the circumvention of the host immune system and the infection of macrophages. This work focused on an important class of surface glycoconjugates, the glycoinositolphospholipids (GIPLs), and their role in the interaction with murine macrophages. GIPLs are expressed on every stage of the parasite life cycle and are the most abundant molecules on its surface. Here we show that these molecules modulate many macrophage functions such as cytokine production, release of nitric oxide and differentially activate MAPK. Although the GIPLs of both New World species are capable of modulating the same mechanisms, they do so to different degrees requiring an examination of their glycan composition. We show that *L. infantum* synthesize mannose rich GIPLs whereas *L. braziliensis* express galactose rich GIPLs.

## Introduction

In the Americas, Leishmaniases are widely distributed from the southern United States to northern parts of Argentina [1]. In Latin America, especially in Brazil, *Leishmania braziliensis* and *Leishmania infantum* are the causative agents of cutaneous (CL) and visceral leishmaniasis (VL), respectively. The severity of the disease may range from self-healing cutaneous ulcers to potentially lethal visceral form [2].

During the life cycle, *Leishmania* parasites have to survive to extreme adverse conditions in both vertebrate and invertebrate hosts [3]. In the vertebrate host, inoculation of metacyclic *Leishmania* promastigotes by the sand fly is followed by neutrophil phagocytosis prior to intracellular differentiation into amastigotes [4]. At the early steps of infection, innate cellular microbicidal mechanisms may include the production of reactive nitrogen intermediates (RNI), reactive oxygen intermediates (ROI) and cytokines (IL-12, TNF-α and IFN-γ) [5], [6]. This is crucial for Th1 polarization and subsequent parasite control in the mouse model. Failure in this process can lead to higher parasite burden and increase severity of disease [7].

To avoid destruction, intracellular parasites must interfere with the cytocidal signaling system of the host. *In vivo* and *in vitro* studies have demonstrated the importance of nitric oxide (NO) production in response to several stimuli such as bacterial lipopolysaccharide (LPS), IFN-γ and TNF-α [8]. It is known that *Leishmania*-infected macrophages fail to activate MAPKs, become less responsive to cytokine stimulation (IL-12 and IFN-γ) [9], [10], [11] and express lower amounts of iNOS and IL-12 [12], [13], impairing T CD4+ cell differentiation to a TH1 phenotype.

The molecular mechanisms involved in the immune system modulation by *Leishmania* have been the focus of many studies. GPI-anchored molecules are closely associated with cell signaling and can act as agonists and second messengers in response to cytokines and other stimuli [9], [14], [15], [16]. The most studied *Leishmania* glycoconjugate is lipophosphoglycan (LPG), whose functions include: attachment and entry into macrophages [17], modulation of NO production [18], inhibition of protein kinase C (PKC) dependent cell activation [19], [20], retardation of phagosome maturation [21], disruption of NADPH oxidase assembly at the phagosome membrane [22], induction of neutrophil extracellular traps (NETs) [23], induction of protein kinase R (PKR) [24], and attachment to the sand fly vector midgut [25]. In *Leishmania*, Toll-like receptor 2 (TLR2) is the main receptor for both LPG and glycoinositolphospholipids (GIPLs), the latter as a less potent agonist [26], [27]. Besides TLR2, *in vivo* studies have also demonstrated the importance of TLR4 and TLR9 during *Leishmania* infection [28], [29], [30].

**Figure 1:**
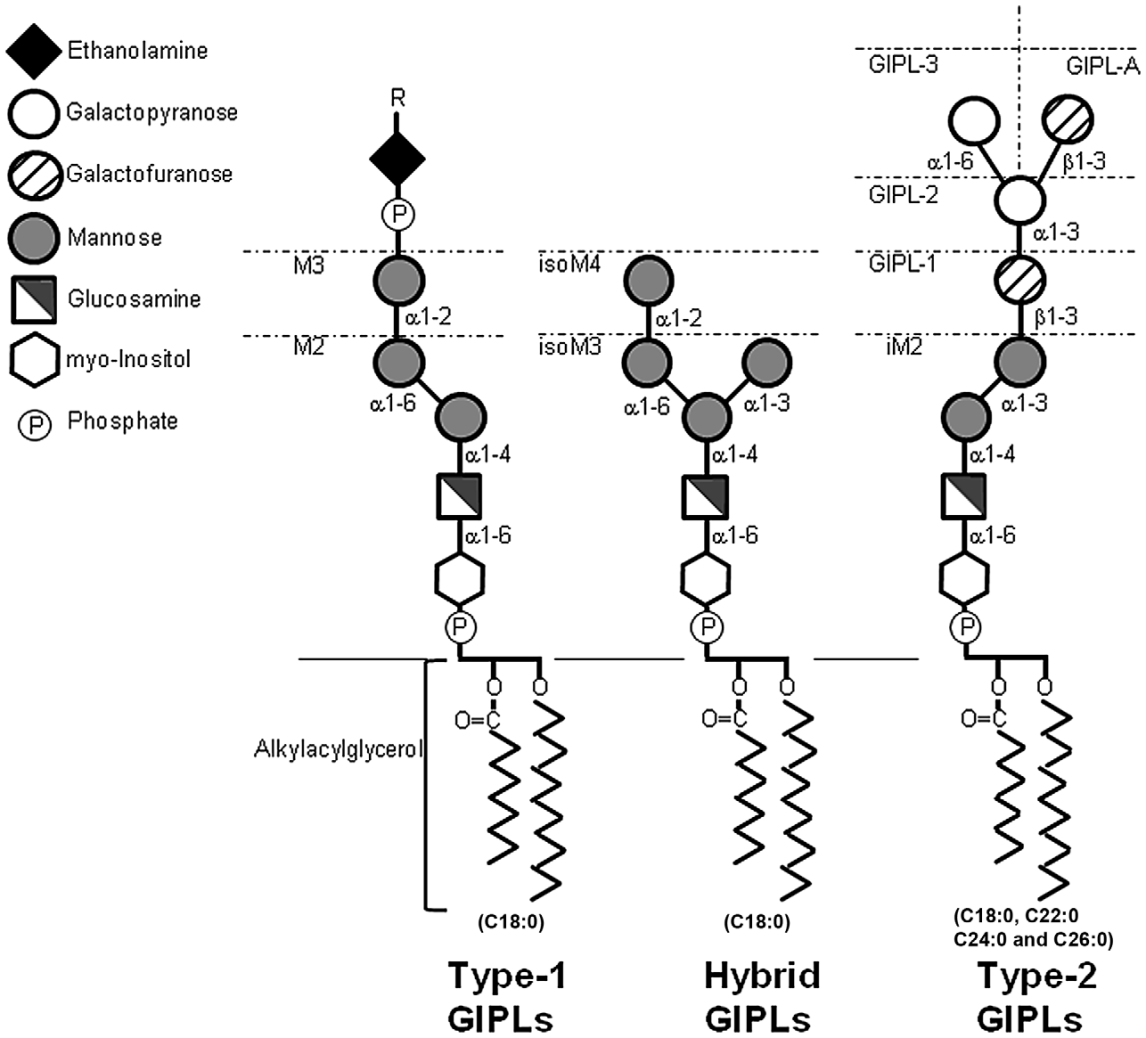
Types of GIPLs. For information on M2, M3, iM2, GIPL-A, isoM3 and isoM4, see introduction. Fatty acid chains vary in different GIPL species: The predominant type fatty acid in Type-1 and Hybrid GIPLs is C_18∶0_, in type-2 GIPLs the predominant lipids are C_18∶0_, C_22∶0_ C_24∶0_ and C_26∶0_. “R” in Type 1 GIPLs represent a protein linked to the GIPL structure by a ethanolamine phosphate residue (e.g. gp63 surface metalloprotease) [31], [78].

Little is known about the functions of GIPLs in *Leishmania* biology, although they are present as the major component of the parasite surface in numbers greater than LPG [31]. The basic GIPL structure is a Manα1-4GlcN linked to an alkyl-acylglycerol through a phosphatidylinositol (PI) residue. Polymorphism in this family of molecules relies on the variety of fatty acid substitutions in the lipid anchor and monosaccharide substitutions in the glycan core moiety, leading to their classification into three groups (Figure 1): Type-I GIPLs are characterized by having an α1,6-mannose residue linked to the Manα1-4GlcN motif. This group is represented by M2 and M3 GIPLs which structures are Manα1-6Manα1-4GlcN-PI and Manα1-2 Manα1-6Manα1-4GlcN-PI. Type I GIPLs are closely related to GPI anchors of proteins with a very homogeneous lipid composition, predominantly C_18∶0_ fatty acids, and are found in Old World species such as *L. donovani*, *L. tropica* and *L. aethiopica* promastigotes [32]. Type-II GIPLs have a much more heterogeneous lipid composition with C_18∶0_, C_22∶0_, C_24∶0_ and C_26∶0_ fatty acids. They can be found in Old World *L. major* [33], [34] and New World *L. mexicana* [35], [36] and *L. panamensis* [36]. Type II GIPLs are characterized by having an α1,3-mannose residue linked to the Manα1-4GlcN motif, similarly to the glycan core of LPG. Structurally, they can range from small iM2 GIPL, Manα1-3Manα1-4GlcN-PI, to longer structures like GIPL-A, Gal_f_β1-3Galα1-3Gal_f_β1-3Manα1-3Manα1-4GlcN-PI and GIPL-3, Galα1-6Galα1-3Gal_f_β1-3Manα1-3Manα1-4GlcN-PI. The third group is the Hybrid-type GIPLs, sharing common features to both Type-I and II with mannose residues located on both C-3 and C-6 positions of the Manα1-4GlcN motif (isoM3 and isoM4). There may be also other substitutions like phosphate sugars and ethanolamine residues [35], [37]. Early studies have shown that GIPLs from *L. major* were highly antigenic, being recognized by sera from chronic CL patients [38]. Recent findings have demonstrated that *L. braziliensis* GIPLs are components of complex membrane microdomains and that these structures were crucial for parasite infectivity and survival [39]. However, little is known about the role of GIPLs in the innate immune compartment, especially in *L. braziliensis* and *L. infantum*.

This work is part of a wider study on the glycobiology of New World species of *Leishmania*. In previous studies, we reported on the LPGs of *L. braziliensis* and *L. infantum* [40], [41] and showed that the differences in LPG structures were relevant in the parasite biology. In this study, we expanded those findings and show the GIPL structures of the two New World Leishmanias also differentially modulate the innate immune system in mouse peritoneal macrophages.

## Materials and Methods

All animals were handled in strict accordance with good animal practice as defined by the Internal Ethics Committee in Animal Experimentation (CEUA) of Fundação Oswaldo Cruz (FIOCRUZ), Belo Horizonte (BH), Minas Gerais (MG), Brazil (Protocol P-0297-06). Knock-out mice handling protocol was approved by the National Commission of Biosafety (CTNBio) (protocol #01200.006193/2001-16).

### Parasites

World Health Reference strains of *L. braziliensis* (MHOM/BR/1975/M2903), *L. infantum* (MHOM/BR/1974/PP75) and *L. donovani* (MHOM/SD/00/1S-2D) were used. Promastigotes were cultured in M199 medium supplemented with 10% heat-inactivated fetal bovine serum (FBS), penicillin 100 units/ml, streptomycin 50 µg/ml, 12.5 mM glutamine, 0.1 M adenine, 0.0005% hemin, and 40 mM Hepes, pH 7.4 at 26°C [40].

### Extraction and purification of GIPLs

Cells were harvested and washed in PBS twice prior to GIPLs extraction with methanol∶chloroform∶water (10∶10∶3). This material was dried under nitrogen stream, resuspended on 0.1 M ammonium acetate buffer containing 5% 1-propanol and loaded onto an octyl-sepharose column (80 ml) equilibrated in the same buffer. The column was subjected to a gradient of 1-propanol in 0.1 M ammonium acetate buffer (5–60%). Three mL fractions were collected and the presence of GIPLs in the fractions was detected by staining aliquots of the fractions on a TLC plate with orcinol∶sulfuric acid (100°C, 5 min) [34]. GIPLs containing fractions were pooled, dried and resuspended in endotoxin-free water (Sanobiol, São Paulo, Brazil). GIPLs concentrations determined as described elsewhere [42]. Prior to use on *in vitro* macrophage cultures, GIPLs were diluted in fresh RPMI.

### Purification of murine peritoneal macrophages and cell culture

Thioglycollate-elicited peritoneal macrophages were removed from BALB/c, C57BL/6 and respective TLR2−/− and TLR4−/− knockouts by peritoneal washing with RPMI and enriched by plastic adherence for 18 h. Cells (3×10^5^ cells/well) were cultured in RPMI, 2 mM glutamine, 50 U/ml of penicillin and 50 µg/mL streptomycin in 96-well culture plates (37°C/5% CO_2_). They were incubated with gamma interferon (IFN-γ) (100 IU/mL) [43], live stationary *Leishmania* parasites (10∶1), GIPLs (1, 5, 10 and 25 µg/mL) and lipopolysaccharide (LPS) (100 ng/mL).

### Cytokine and nitrite measurements

For CBA multiplex cytokine detection, cells were plated as described above for 1 h before washing with RMPI without serum. RPMI supplemented with 10% FBS was added with (for primed macrophages) or without (for non-primed macrophages) the addition of IFN-γ (3 IU/mL) [44] and incubated for 18 h (37°C, 5% CO_2_). GIPLs (25 µg/mL) and LPS (100 ng/mL) were added and incubated for 48 h. Supernatants were collected and stored at −70°C and cytokines (IL1-β, IL-2, IL-4, IL-5, IL-10, IL-12p40, IFN-γ and TNF-α) were determined using the BD CBA Mouse Cytokine assay kits according to the manufacturer's specifications (BD Biosciences, CA, USA). Flow cytometric measurements were performed on a FACS Calibur flow cytometer (Becton Dickinson, Mountain View, CA). Cell-Quest™ software package provided by the manufacturer was used for data acquisition and the FlowJo software 7.6.4 (Tree Star Inc., Ashland, OR, USA) was used for data analysis. A total of 1,800 events were acquired for each preparation. Results are representative of two experiments in duplicate.

For inhibition studies, cell suspensions were washed with RPMI and enriched by plastic adherence for 18 h as described above without the addition of IFN-γ. Cells were pre-incubated with GIPLs (15 min) prior to stimulation with LPS or IFN-γ. Supernatants were collected after 24 h for NO, TNF-α and IL-12 measurements. When used, LPS or IFN-γ were added 15 min after the addition of GIPLs. Culture supernatants were collected and nitrite concentrations determined by Griess reaction [45] and TNF-α and IL-12 concentrations were determined using ELISA (BD). Results are representative of two experiments in triplicate.

### PI-specific phospholipase-C treatment (PI-PLC)

To evaluate whether intact GIPL structure is required for activity. Purified GIPLs were ressuspended in 150 µl CHAPS buffer (298 mg HEPES, 47 mg EDTA and 50 mg CHAPS in 50 ml endotoxin-free water) and 2 U of PI-PLC (Sigma) (37°C, 16 h). Peritoneal macrophages were plated and stimulated with intact and PI-PLC treated GIPLs as described above. Nitrite content was measured on the supernatants by Griess reaction [45].

### Preparation of cell lysates and immunoblotting

Stimulated cells (3×10^6^/sample) were washed with ice-cold PBS, lysed in lysis buffer (20 mM Tris-HCl pH 7.5, 1% Triton X-100, 1 mM sodium orthovanadate, 1 mM phenylmethylsulfonyl fluoride (PMSF), 50 mM sodium fluoride, 150 mM NaCl, 5 mM ethylenediamine tetraacetic acid (EDTA), 10% Glycerol (v/v), 0.5 mM dithiothreitol (DTT) and protease inhibitor cocktail from Sigma®). Cells were harvested with a plastic scraper and centrifuged at 13,000× *g* (4°C, 10 min). Supernatants were transferred to fresh tubes and stored at −20°C until used. Cell lysates were resolved by SDS-PAGE, transferred to a nitrocellulose membrane and blocked (5% milk in TBS-0.1% Tween 20) for 1 h. Primary Abs (anti dually phosphorylated ERK, dually phosphorylated p38 and Total ERK, 1∶1,000) were incubated for 16 h at 4°C. Membranes were washed (3×10 min) with TBS-0.1% Tween 20 and incubated 1 h with anti-mouse IgG conjugated with peroxidase (1∶10,000). The reaction was visualized using luminol.

### Nitrous acid deamination

Purified GIPLs were delipidated by nitrous acid deamination (300 µl of 0.5 M sodium acetate and 300 µl of 0.5 M NaNO_2_) for 16 h at 37°C [40]. Samples were dried, resuspended in 0.1N HAc/01M HCl and applied to a phenyl-sepharose column (1 mL). The sugar headgroups were eluted using 0.1N HAc/0.1M HCl. After washing column with 2 volumes of water, lipids and unreacted GIPLs were eluted using Solvent E (H_2_O/ethanol/diethyl ether/pyridine/NH_4_OH; 15∶15∶5∶1∶0.017) [46].

### Gel filtration

To desalt, deaminated GIPLs glycan headgroups were applied to Sephadex G-25 (1×5 cm) columns equilibrated with 10 ml of water. Eluted deaminated glycan headgroups were collected in 0.5 ml fractions, checked for the presence of salt using silver nitrate and dried in Speed-Vac [40].

### Strong acid hydrolysis

To obtain depolymerized neutral monosaccharides, deaminated glycan headgroups were subjected to strong acid hydrolysis (2N trifluoracetic acid, 3 h, 100°C) and dried in Speed-Vac. To remove acid, 500 µl of toluene were added to samples, homogenized using vortex and evaporated twice under N_2_. Samples were resuspended in 500 µl of water and desalted by ion exchanging chromatography.

### Ion exchange chromatography

To remove salt from neutral monosaccharides, dried depolymerized neutral monosaccharides were diluted in 500 µl of H_2_O and applied onto a column containing AG1-X8 acetate form over AG50W-X12 resins. Samples were eluted with 5 mL of water and dried in a Speed-Vac instrument [47].

### Thin layer chromatography (TLC)

Intact and deaminated GIPLs were chromatographed on TLC Silica Gel 60 plates (Merck). To compare rough GIPL content of *L. braziliensis*, *L. infantum* and as reference *L. donovani*. Intact GIPLs were chromatographed in 1-butanol∶methanol∶water (4∶4∶3 v/v) for 20 h. To access Deamination by nitrous acid sensitivity, GIPLs were subjected to nitrous acid deamination as described above and resolved in chloroform∶methanol∶13M ammonium hydroxide∶1M ammonium acetate∶water (180∶140∶9∶9∶23 v/v) for 20 h. Bands were visualized as described above [46], [48].

### Fluorophore-assisted carbohydrate electrophoresis (FACE)

To access the oligosaccharide composition, deaminated GIPLs headgroups were fluorescently labeled with 0.05 N ANTS (8-aminonaphthalene-1,3,6-trisulfate) and 1 M cyanoborohydride (37°C, 16 h). To determine the monosaccharide composition of the GIPLs, depolymerized and desalted monosaccharides were fluorescently labeled with 0.1 M AMAC (2-aminoacridone) in 5% acetic acid and 1 M cyanoborohydride. Labeled sugars were subjected to FACE and the gel was visualized under UV light. Oligoglucose ladders (G_1_–G_7_) and monosaccharides (D-galactose, D-glucose and D-mannose) (Sigma) were used as standards for oligosaccharides and monosaccharide gels, respectively [47], [49].

### HPLC

Desalted monosaccharides were separated using a DX-500 HPLC (Dionex Corp.) with ED40 electrochemical detection. Samples were run on a CarboPac PA10 column (4×250 mm) in the presence of 18 mM NaOH (flow rate 1 mL/min, 2000 psi). D-galactose, D-glucose and D-mannose (100 µg/mL) were used as standards.

### Statistical analyses

For nitrite and cytokine measurements, the Shapiro–Wilk test was conducted to test the null hypothesis that data were sampled from a Gaussian distribution [50]. The P value (P>0.05) showed that data did not deviate from Gaussian distribution. For this reason, student's “t” test and ANOVA were performed to test equality of population medians among groups and independent samples. Data were analysed using GraphPad Prism 5.0 software (Graph Prism Inc., San Diego, CA) and P<0.05 was considered significant.

## Results

### Nitrite and cytokine production

**Figure 2:**
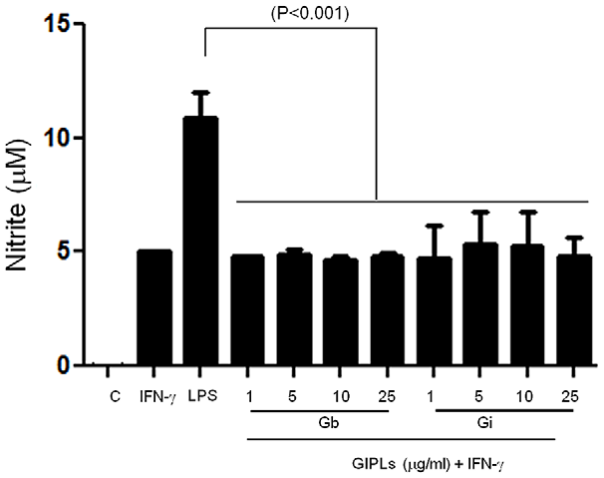
Nitrite production by BALB/c primed macrophages after stimulation with different concentrations of GIPLs. C, negative control; IFN-γ, gamma-interferon; LPS, lipopolysaccharide; Gb, *L. braziliensis* GIPLs; Gi, *L. infantum* GIPLs. Cells were primed with IFN-γ (3 IU/ml) for 18 h prior to the addition of the GIPLs or LPS (positive control). Non primed cells and primed cells without the addition of a new stimulus were also used as controls. ANOVA test was performed and P<0.05 was considered significant. Results are the representation of three experiments in triplicate.

**Figure 3:**
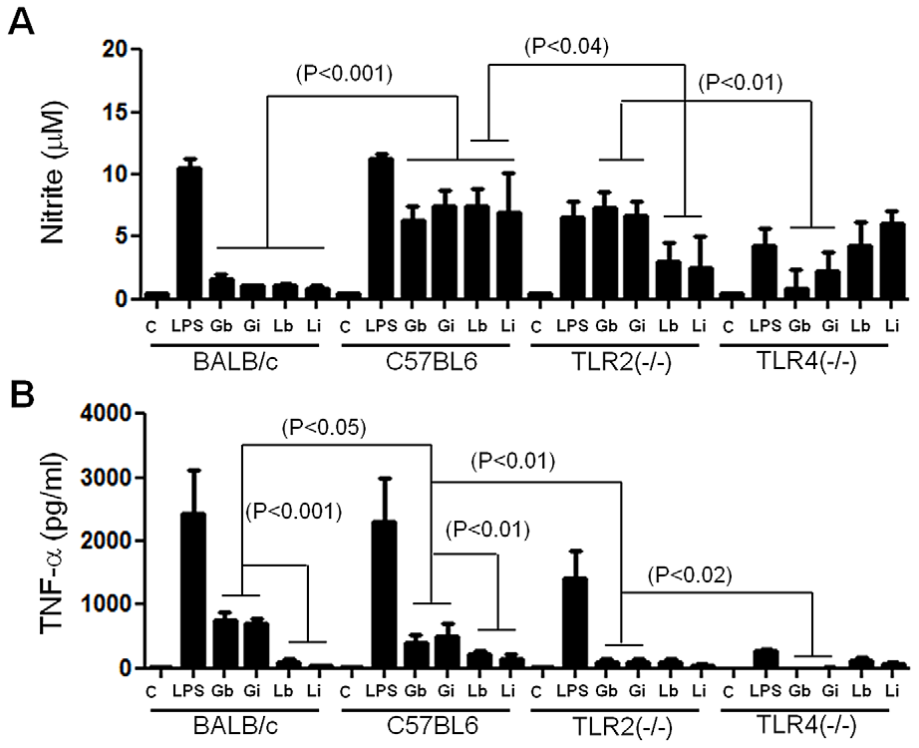
Nitrite and TNF-α production by primed macrophages after stimulation with GIPLs and parasites. C, negative control; Gb, *L. braziliensis* GIPLs; Gi, *L. infantum* GIPLs; Lb, *L. braziliensis* live promastigotes and Li, *L. infantum* live promastigotes. Cells were pre-incubated with IFN-γ (3 IU/ml) for 18 h then 25 µg/mL of GIPLs or 100 ng/mL of LPS was added. Supernatants were collected 48 hours later, in (**A**) NO concentrations were measured by Griess reaction and in (**B**) TNF-α concentrations determined by flow cytometry. ANOVA test was performed and P<0.05 was considered significant.

To determine whether GIPLs from both *L. braziliensis* and *L. infantum* are able to induce the production of nitrite, peritoneal macrophages were incubated with live promastigotes (10∶1) or treated with different concentrations of GIPLs (1 to 25 µg/mL) with IFN-γ serving as positive control (100 IU/mL). Neither of the purified GIPLs could induce any detectable increase in the production of nitric oxide (NO) in primed BALB/c macrophages (Figure 2) nor the production of the cytokines tested (IL1-β, IL-2, IL-4, IL-5, IL-10, IL-12p40, IFN-γ and TNF-α) in non-primed macrophages in all other mice lineages (data not shown). No NO production was detected in non-primed macrophages of BALB/c, C57BL/6, TLR2−/− and TLR4−/− mice (data not shown) and in BALB/c primed macrophages (Figure 3A). A higher NO production was detected on C57BL/6 IFN-γ-primed macrophages stimulated with GIPLs and live promastigostes when compared to BALB/c mice (P<0.001). There was a significant NO production in primed C57BL/6 and TLR2 (−/−) macrophages stimulated with GIPLs in comparison to TLR4 (−/−) (P<0.01) (Figure 3A) suggesting the involvement of TLR4 in this activation. Also, a slight reduction of NO production was noticed in macrophages from TLR2 (−/−) mice stimulated with live promastigotes when compared to C57BL/6 (P<0.04). This reduction may indicate the participation of other parasite molecules that are recognized by TLR2 such as the LPG. The LPG is known to be a potent agonist of TLR2 and is capable of inducing the production of cytokines (IL-12, IFN-γ and TNF-α) in macrophages and NK cells [26], [27]. Differently from NO, TNF-α production was higher in BALB/c mice than in C57BL/6 (P<0.05) in response to the stimulation of GIPLs from both species. Similarly this production was higher in TLR2 (−/−) than TLR4 (−/−) (P<0.02). This data also indicate a slight TLR4 involvement in TNF-α production. In both WT macrophages, the TNF-α production was higher after stimulation with GIPLs in comparison to live promastigotes (Figure 3B) (P<0.01). A lower TNF-α production was noticed in TLR2 (−/−) suggesting the involvement of TLR2 in this process.

GIPLs did not induce the production of any of the cytokines tested (IL1-β, IL-2, IL-4, IL-5, IL-10, IL-12p40 and IFN-γ) in BALB/c, C57BL/6, TLR2 (−/−) and TLR4 (−/−) mice (data not shown). In all experiments, live parasites from both species induced cytokine production close to background levels (Figure 3B and data not shown). These results suggest that GIPLs are able to activate NO in C57BL/6 mice and TNF-α in either BALB/c or C57BL/6 during the early steps of infection, and were not able to stimulate most of the cytokines assayed.

### Inhibition of nitrite and IL-12 production in BALB/c macrophages pre-exposed to GIPLs

**Figure 4:**
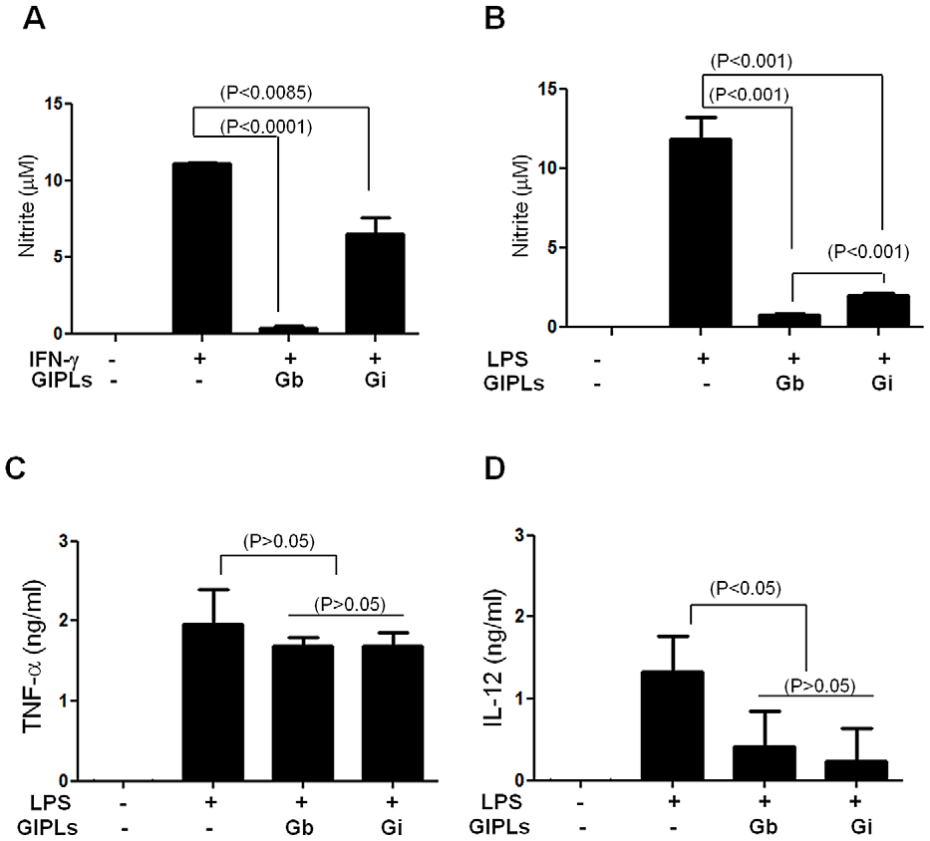
Modulation of nitrite, TNF-α and IL-12 production by *Leishmania* GIPLs in BALB/c macrophages. Cells were incubated with GIPLs (25 µg/ml) from *L. braziliensis* (Gb) *and L. infantum* (Gi) for 15 min prior to stimulation with IFN-γ (100 IU/ml) (A) or LPS (100 ng/mL) (B). Nitrite content was measured by Griess reaction; TNF-α and IL-12 concentrations were measured by ELISA. P<0.05 was considered significant. Results are the representation of three experiments.

Compared to LPG, GIPLs had a less potent agonistic activity to stimulate nitrite and cytokine production in previous studies [27]. To test if this pattern was due to inhibition and/or lack of activation, thioglycollate elicited peritoneal macrophages were pre-incubated with GIPLs prior to stimulation with IFN-γ or LPS. A strong inhibition (aprox. 42%) of NO production stimulated by IFN-γ was observed for *L. infantum* GIPLs and was almost completely abolished for *L. braziliensis* (P<0.01) (Figure 4A). A similar response was observed for LPS and this inhibition was more pronounced in *L. braziliensis* (P<0.001) (Figure 4B). Pre-incubation with GIPLs was also able to inhibit approximately 65% of IL-12, but not TNF-α production (Figures 4C and D). These results indicate an inhibitory role of GIPLs.

**Figure 5:**
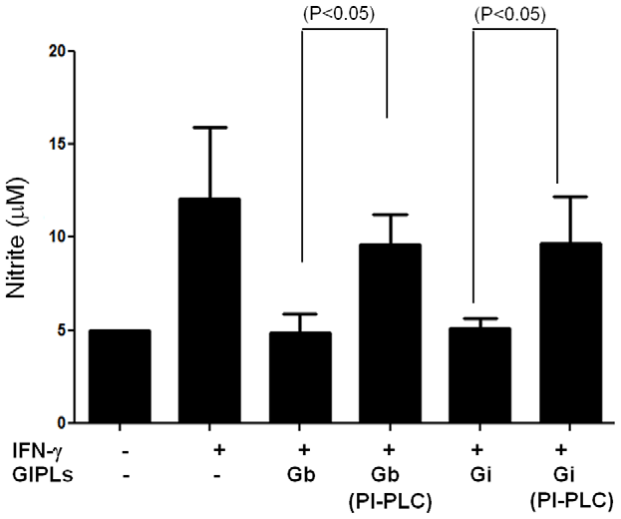
Modulation of nitrite production by macrophages stimulated with intact and PI-PLC treated GIPLs. Mouse peritoneal macrophages were incubated with GIPLs (25 µg/ml) from *L. braziliensis* (Gb), *L. infantum* (Gi), PI-PLC treated *L. braziliensis* GIPLs (Gb PI-PLC) and *L. infantum* PI-PLC treated GIPLs (Gi PI-PLC) for 15 min prior to stimulation with IFN- γ (100 IU/ml). Nitrite content was measured by Griess reaction on the supernatants after 24 h. Student “t” test was performed and P<0.05 was considered significant. Results are the mean of two experiments.

Also, to test whether the intact structure of GIPLs is required for its inhibitory activity Macrophages were incubated with intact and PI-PLC treated GIPLs. As shown on Figure 5 PI-PLC treated GIPLs failed to inhibit NO production by IFN-γ stimulated cells.

### Activation of MAPKs

**Figure 6:**
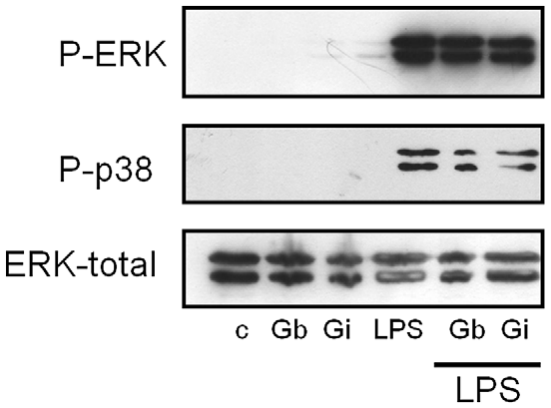
Activation of MAPKs (ERK and p38) by *Leishmania* GIPLs in BALB/c peritoneal macrophages. Mouse peritoneal macrophages were stimulated for 30 min with 25 µg/mL of GIPLs. Dually phosphorylated MAPKs were detected by western blot. C, negative control; Gb, *L. braziliensis* GIPLs and Gi, *L. infantum* GIPLs. Also cells were incubated with GIPLs prior to stimulation with LPS; total ERK content as a normalizing protein.

Since GIPLs were strong inhibitors of cytokine production, we investigated whether those molecules could modulate MAPKs activation. Mouse peritoneal macrophages were previously incubated with GIPLs and MAPK activation was detected using western blot. No significant activation of p38 and only a minimal induction of ERK were observed. Also when cells were preincubated with GIPLs prior to stimulation with LPS, there was a reduction on the phosphorylation of both ERK and p38 (Figure 6). Densitometer analysis normalized by total-ERK expression detected an 18% and 17.5% decrease on ERK activation for *L. braziliensis* and *L. infantum*, respectively. For p38 this inhibition was 16.5% and 33%, respectively.

### Preliminary characterization of *L. braziliensis* and *L. infantum* GIPLs

**Figure 7:**
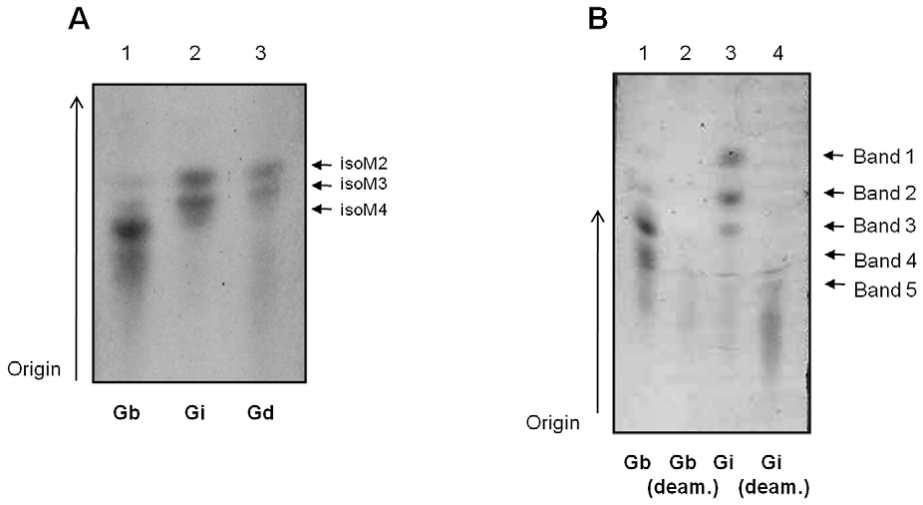
Thin layer chromatography (TLC) of *Leishmania* glycoinositolphospholipids (GIPLs). (A) Purified intact GIPLs: Lane 1, *L. braziliensis* GIPLs (Gb); lane 2, *L. infantum* GIPLs (Gi) and lane 3, *L. donovani* GIPLs (Gd). The assignments for *L. donovani* structures are: isoM2 as Manα1-3Manα1-4GlcN-PI; isoM3 as Manα1-6(Manα1-3)Manα1-4GlcN-PI and isoM4 as Manα1-2Manα1-6(Manα1-3)Manα1-4GlcN-PI [32]. LPG, lipophosphoglycan; GPI, glicosyl phosphatidylinositol. (B) Deaminated GIPLs. Lane 1, *L. braziliensis* untreated GIPLs (Gb); lane 2, deaminated *L. braziliensis* GIPLs (Gb deam.); lane 3, *L. infantum* untreated GIPLs (Gi) and Lane 4, deaminated *L. infantum* GIPLs (Gi deam.).

Due to the interspecific differences in the intensity of NO and IL-12 production inhibition (Figures 4) and MAPKs activation (Figure 6), we examined whether those variations could be due to polymorphisms in GIPLs structure and composition. Intact GIPLs were resolved on TLC plates and the GIPL profile differed between the two species (Figure 7A). *Leishmania braziliensis* exhibited slower migrating GIPLs compared to *L. infantum*, whose profile was very similar to *L. donovani* [32] with three main bands co-migrating with isoM2, isoM3 and isoM4. In *L. braziliensis*, the three faster bands co-migrated with bands isoM2, isoM3 and isoM4 of *L. donovani*. All bands were susceptible to nitrous acid deamination, and this is consistent with the presence in the GIPLs of a non-N-substituted glucosamine residue (Figure 7B), a hallmark of *Leishmania* GIPLs anchors [51].

**Figure 8:**
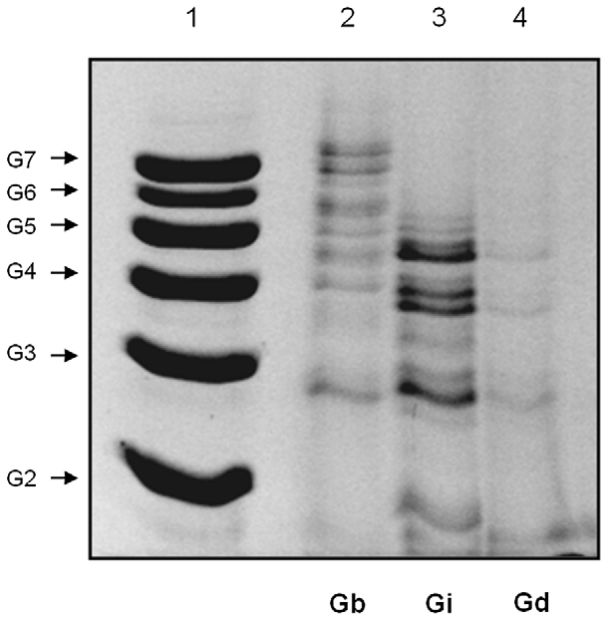
Fluorophore-assisted carbohydrate electrophoresis (FACE) of *Leishmania* GIPLs. Lane 1, oligoglucose ladder represented by G2-G7; lane 2, *L. braziliensis* GIPLs (Gb) and lane 3, *L. infantum* GIPLs (Gi) and lane 4, *L. donovani* GIPLs (Gd).

To better determine sizes of the glycan portions, purified GIPLs were deaminated and desalted. The carbohydrate portions were reductively labeled with a fluorphore and then subjected to FACE. Consistent with the TLC data (Figure 7), the carbohydrate portions of the GIPLs from *L. braziliensis* were larger exhibiting up to 8–9 sugars while those from *L. infantum* and *L. donovani* consisted of up to 4–5 sugars (Figure 8).

**Figure 9:**
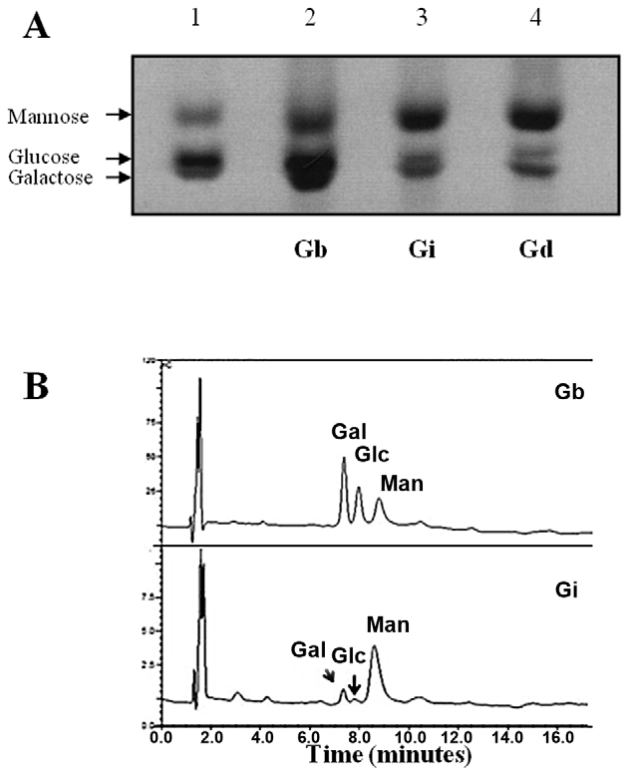
Monosaccharide profile of *Leishmania* glycoinositolphospholipids (GIPLs). (A) Fluorophore-assisted carbohydrate electrophoresis (FACE). Lane 1, standards represented by galactose, glucose and mannose (100 µg/ml); lane 2, *L. braziliensis* GIPLs (Gb); lane 3, *L. infantum* GIPLs (Gi) and Lane 4, *L. donovani* GIPLs (Gd). (B) High performance liquid chromatography (HPLC). Gal, galactose; Glc, glucose and Man, mannose.

To access sugar composition, GIPLs were subjected to strong acid hydrolysis and the resulting monosaccharides were analysed by FACE and HPLC (Figure 9A and B). Consistent with the TLC data (Figure 7A), the monosaccharide composition of *L. infantum* GIPLs was very similar to the GIPLs from *L. donovani* (Figure 9A). The relative amounts of galactose, glucose and mannose (calculated by the relative peak areas on HPLC) were determined (Figure 9B). Supporting our other findings and GIPL assignments, the GIPLs from *L. infantum* had higher concentrations of mannose (82%), followed by galactose (12%) and glucose (6%). This indicates that these are mostly Type I or hybrid GIPLs, whose structure bears a terminal mannose, but a small proportion of Type II GIPLs (terminated in galactose) is probably present. On the other hand, *L. braziliensis* GIPLs had higher galactose content (42%), followed by, mannose (30%) and glucose (28%), thus suggesting a Type II GIPL structure.

## Discussion

Infection with protozoan parasites remains a prominent problem in different parts of the world having a major impact on public health in the developing countries. Leishmaniases are considered by World Health Organization [52] as one of the major six important infectious diseases worldwide. This class of parasitic diseases currently affects over 12 million people all around the world, up to 1.5 million new individuals developing the visceral and tegumentar disease respectively each year. In Brazil, most of those cases are caused by *L. infantum* and *L. braziliensis*, respectively.

The question of how parasites interact with hosts cells to promote infection and survival has been the focus of interest for a long time. In order to survive in the macrophage cells, *Leishmania* has to prevent or inhibit a variety of intracellular mechanisms of parasite killing, one of which is dependent on ROS and RNI [53], [54]. However, RNI alone is effective for controlling visceral Leishmaniasis [55].

Parasite surface molecules, especially the LPG, have long been known to play an important role in the host parasite interactions [17], [27], [56]. In this work, we focused on another class of glycoconjugates, the GIPLs in two New World species of *Leishmania* with different known immunopathologies. These molecules are abundantly present on the parasite surface in numbers great that 10^7^. Recently, they have been found associated to lipid rafts, essential for parasite infectivity and selective modulation of the host cell response [39]. In fact, there are several indications that GIPLs and other GPI-anchored molecules participate in cell signaling and are involved in the assembly of the NADPH oxidase complex, NO production [16], [57], [58], [59], [60] and inhibition of LPS and TNF-α induced c-*fos* gene expression by macrophages [61]. Also synthetic LPG, whose GPI anchor is structurally similar to GIPLs, can stimulate ERK activation and therefore inhibit IL-12 synthesis by macrophages [9].

Previous studies have demonstrated GIPLs antigenicity in chronic patients infected with *L. major* [38], [62]. However, information concerning the biological relevance of GIPLs at early steps of infection in the innate immune compartment was still limited. Here, we demonstrated that GIPLs from both New World species were not able to activate the production of NO in non-primed macrophages, which was similar to published data from Old World species [59], [60]. In primed macrophages an initial NO and TNF-α production was detected. Further, GIPLs differentially inhibited NO production even in the presence of IFN-γ and LPS, two major NO inducers. Previous studies indicated that LPG was a more potent agonist than GIPLs for the induction of pro-inflammatory cytokines [26], [27]. In general, in comparison to LPS, GIPLs induced a lower production of NO and TNF-α. Also, they exhibited a strong inhibitor pattern during NO and cytokine induction, especially IL-12.

Similar strategy was demonstrated using crude extracts of the rat tapeworm *Hymenolepis diminuta*, although using different pathways. As shown by Johnston *et al.* (2010) [63], crude extracts of this tapeworm could inhibit the production of TNF-α and IL-6 by mouse and human macrophages stimulates with TLR agonists poly(I:C) and *Flagellin*. These extracts also protected mice from experimental colitis accompanied by enhanced IL-10 and IL-4 production.

*In vivo* studies using Old World species of *Leishmania* have demonstrated the importance of TLRs and other components of the innate immune system during infection. MyD88 is the most common adaptor molecule for the activation of NF-κB in most TLRs [28]. Also many studies using gene knockout have shown the importance of TLR and MyD88 adaptor molecule for cytokine production [29], IL-1 promoter activation [64], IFN-γ and IL-12 production [65].

NF-κB activation through TLR2 [26], elastase dependent neutrophil control of *L. amazonensis* promastigotes [66], and ultimately parasite control and lesion healing [27], [65], [67].

Indeed, in primed macrophages, GIPLs from both New World species were able to stimulate the production of NO, and this induction was mostly via TLR4 and to a lesser extent TLR2 (Figure 3A). However, no difference was observed while stimulating with live parasites.

Interestingly, in the *L. braziliensis* model, the TLR2 receptor plays a much more regulatory role in dendritic cells, repressing IL-12p40 and promoting IL-10 expression. This observation is correlated with sustained IFN-γ production and enhanced parasite control in TLR2 (−/−) mice [68]. However, in macrophages exposed to GIPLs, this difference in NO expression between TLR2 (−/−) and TLR4 (−/−) strains was not due to IL-12, IFN-γ or IL-10 production (Figures 3B and 4). Also this induction was more pronounced in C57BL/6 than in BALB/c this was expected since C57BL/6 derived macrophages tend to be more responsive to stimuli than BALB/c macrophages [69]. These data are in accord with previous studies showing that related GIPLs from *Trypanosoma cruzi* are able to activate TLR4 [70] and studies with Old World species of *Leishmania* being able to activate TLR2, TLR3, TLR4 and TLR9 [28]. With exception to TNF-α, GIPLs and live parasites from *L. braziliensis* and *L. infantum* were not able to induce the other cytokines studied (IL-1β, IL-2, IL-4, IL-5, IL-10, IL-12p40 and IFN-γ) in primed and non-primed macrophages (data not shown). Thus, we conclude that the GIPLs from these two New World species are less potent agonists or strong inhibitors for macrophages and the data presented here supports that the later might be true.

When pre incubated with GIPLs, a strong inhibition of both NO and IL-12 production was observed (Figures 4C and D). This inhibitory effect seems to be in specific pathways since no significant inhibition was detected for TNF-α (Figure 4C). This inhibition is dependent on the intact structure of GIPLs since PI-PLC digested GIPLs that have its glycan core detached from its lipid anchor, failed to inhibit NO production by IFN-γ stimulated macrophages (Figure 5). Also, regarding TNF-α, only WT mice were able to trigger the production of this cytokine and this production was very low for TLR2 (−/−) and completely absent in TLR4 (−/−) (Figure 3B). These data supports the premise that NF-κB translocation is not affected by GIPLs exposure [71]. It is noteworthy that the inhibition of IL-12 is not due to production of IL-10, because we observed no IL-10 production either in unprimed (data not shown) or in primed macrophages incubated with GIPLs (Data not shown).

In TLR signaling, the most common adaptor molecule is MyD88 but other adaptor molecules may be involved in NF-κB translocation such as mitogen-activated protein kinases (JNK or p38) [72]. Early studies showed that the *Leishmania* LPG can inhibit IL-12 without affecting NF-κB translocation to the nucleus [9]. For maximal downstream activation and GPI-induced gene expression, a full activation and cooperation Protein Tyrosine Kinase (PTK) and Protein Kinase C (PKC) are required. Although iM4 *L. mexicana* GIPL stimulated rapid PTK phosphorylation it failed in activating PKC [16]. In fact the unusual glycolipid composition (mostly alkyl-acyl-glycerol) of *Leishmania* GIPLs inhibits the activations of PKC [58], [73]. This is in accordance with our observations that GIPLs not only fail on inducing a pro-inflammatory response in non-macrophages but also that the GIPLs inhibit the productions of IL-12 and NO.

Also we tested whether GIPLs from both New World species were able modulate the phosphorylation of MAPKs. We observed that the GIPLs activate only ERK, whereas LPS activated both ERK and p38 (Figure 6). Also we observed that the GIPLs can prevent the phosphorylation of both ERK and p38 MAPKs stimulated by LPS. However, ERK activation was too low to provide evidence for any further effect on IL-12 production. It is likely that *L. braziliensis* and *L. infantum* GIPLs have a profound effect on macrophage cell signaling affecting PTKs, PKCs and MAPKs, and that GIPLs from both species use similar pathways but differ in the intensity in which they modulate NO and IL-12 production.

In this work, GIPLs interacted with primed macrophages resulting only in the production of NO and TNF-α. GIPLs are abundant in the amastigote stage of *Leishmania* and are associated to highly specialized microdomains [39] and the participation of each kind of GIPL on the process is still under debate [74], [75], [76]. Also it is possible that the dependency on a particular glycolipid may vary throughout species and life cycle stage. The data presented here clearly supports the hypothesis that *Leishmania* GIPLs, differently from other trypanosomatids, may contribute to build a safer environment to promote infection by manipulating macrophage function and by disrupting the polarization of TH1/TH2 response, through inhibiting IL-12 production during the initial stages of infection and manipulate macrophage for parasite survival.

In general, LPGs and GIPLs share similar lipid anchor moieties among the various species of *Leishmania* and the integrity of this portion is important for TLR2 activation [27]. To ascertain if the differences in the inhibition of NO and IL-12 production could be related to polymorphisms in GIPL structure, we analyzed the carbohydrate core of *L. braziliensis* and *L. infantum* GIPLs. Previous studies from our group showed that the phosphoglycan domains of LPGs from *L. braziliensis* and *L. infantum* differ in structure and composition [40], [41] and differences in glycan portions of GIPLs were also observed in this study. The iM2 species of GIPLs possesses the structure Manα1-3Manα1-4GlcN-P) similar to LPG core region, and isoM3 has a hybrid glycan in GIPLs (substitutions on both the third and sixth carbons of the distal mannose) with the structure of Manα1-6(Manα1-3Manα1-4GlcN-PI. Our structural observations indicated that the GIPLs from *L. infantum* are similar to the known structures in *L. donovani* [32] and are composed mainly of mannose residues. This data suggests that the majority of these GIPLs as Type I GIPLs and Hybrid GIPLs. On the other hand, *L. braziliensis* GIPLs shows a different profile of sugar composition and different bands distinguishable on TLC (Figure 7A). We determined that there was a stoichiometric ratio of galactose and mannose in the glycan portion of these GIPLs. This data suggest that these GIPLs are similar to the closely related species *L. panamensis* [36], which have a common Gal_f_β1-3Manα1-3Manα1-4GlcN-myoinositol glycan headgroup and a structurally related to LPG lipid anchor, suggestive of Type II GIPLs. Type II GIPLs can be very diverse and substitutions on the 3^rd^ carbon of the Gal_f_ residue by Galα-1, Galα1-3galα1, and even longer saccharides like Manα1-PO_4_-6Galα1-6Galα1 can be detected in other species like *L. major* [31]. These substitutions can lead to large GIPLs containing up to 7, 8 or even more hexoses [34], [36], which we observed from the *L. braziliensis* GIPLs as seen on Figure 8.

In conclusion, GIPLs from both New World species *L. infantum* and *L. braziliensis* have a strong inhibitory potential during intracellular *Leishmania* infection of the mammalian host. Only an initial production of NO and TNF-α was detected after stimulation by GIPLs. Due to their importance in modulating NO and cytokine production, these molecules could be possible targets to alternative immunological and chemotherapeutic control methods. The preliminary qualitative analysis of GIPLs from these two species showed that they differ in composition and structures thus, suggesting that the structural distinctions could be responsible for differential NO and IL-12 inhibition in macrophages. Also, GIPLs were also capable of affecting macrophage ability to produce NO in the presence of IFN-γ and LPS. These data, together with already published data from other groups, suggest that GIPLs may be involved in the interaction with the macrophage triggering a minimal pro-inflammatory response in the host and to the benefit of the parasite. Glycoconjugate interspecies polymorphisms, not only in the GIPLs, but also in LPG, gp63 and other GPI-anchored molecules could be important for differential establishment of infection. These polymorphisms could result in different clinical outcomes, such as those shown by *L. infantum* and *L. braziliensis*, causative agents of a visceral and tegumentary forms, respectively [77].
